# Splice-acceptor site mutation in *p53* gene of hu888 zebrafish line

**DOI:** 10.1007/s13353-014-0239-4

**Published:** 2014-09-03

**Authors:** Alicja Piasecka, Paweł Brzuzan, Maciej Woźny, Sławomir Ciesielski, Dariusz Kaczmarczyk

**Affiliations:** Department of Environmental Biotechnology, Faculty of Environmental Sciences, University of Warmia and Mazury in Olstzyn, ul. Słoneczna 45G, 10-709 Olsztyn, Poland

**Keywords:** Alternative splicing, hu888, Splice-site mutation, Tumor suppressor p53, Zebrafish

## Abstract

The p53 transcription factor is a key tumor suppressor and a central regulator of the stress response, which has been a subject of intense research for over 30 years. Recently, a zebrafish line which carries splice site mutation (G>T) in intron 8 of *p53* gene (*p53* ^*hu888*^), encoding the p53 paralogue, was developed (The Zebrafish Mutation Project). To uncover molecular effects of the mutation, we raised hu888 zebrafish line to adulthood and analyzed DNA, mRNA data, and protein levels of p53 to assess their potential contribution in molecular mechanisms of the mutant fish. To obtain zebrafish individuals homozygous for the point mutation, *p53* ^*hu888*^ carriers were repeatedly incrossed but only heterozygous mutants (*p53* ^*hu888/+*^) or p53-wild type hu888 zebrafish (*p53* ^*+/+*^) were identified in their progeny. By evaluation of *p53* expression changes in the liver of mutant and wild type hu888 zebrafish as well as of Tübingen reference strain, we demonstrated that two types of splicing occurred in each case: a classical one and the alternative splicing which involves the activation of cryptic splice-acceptor site in the exon 9 of zebrafish p53 pre-mRNA. The alternative splicing event results in a deletion 12 nucleotides in the mature mRNA, and produces a shortened variant of p53 protein. Interestingly, expression of p53 protein in liver of both heterozygous and wild type hu888 zebrafish was highly reduced compared to that in the reference strain.

## Introduction

The growing number of forward and reverse genetic tools that are available for zebrafish have successfully identified and characterized many genes involved in development and diseases. Functional knockout lines have expanded the experimental power of the zebrafish genome to provide insight into how orthologous mammalian genes function in similar processes (Goodale et al. 2012). One of the most extensively studied genes is the *p53* tumor suppressor gene. Since its discovery in 1979, the role of p53 protein in cancer has been studied intensively (Levine and Oren 2009). The p53 protein acts primarily as a transcription factor that regulates the expression of many different genes in response to a wide variety of stress signals (Beckerman and Prives 2010; Lane 1992). The significance of *p53* in cancer is underscored by the fact that it is the most frequently mutated tumor suppressor gene in human cancers, with more than half of solid tumors harboring mutations in the *p53* locus (Olivier et al. 2010). All mutations in the *p53* gene found in human cancers are compiled by the International Agency for Research on Cancer (IARC TP53 Database, http://www-p53.iarc.fr/) (Petitjean et al. 2007).

**Fig. 1:**
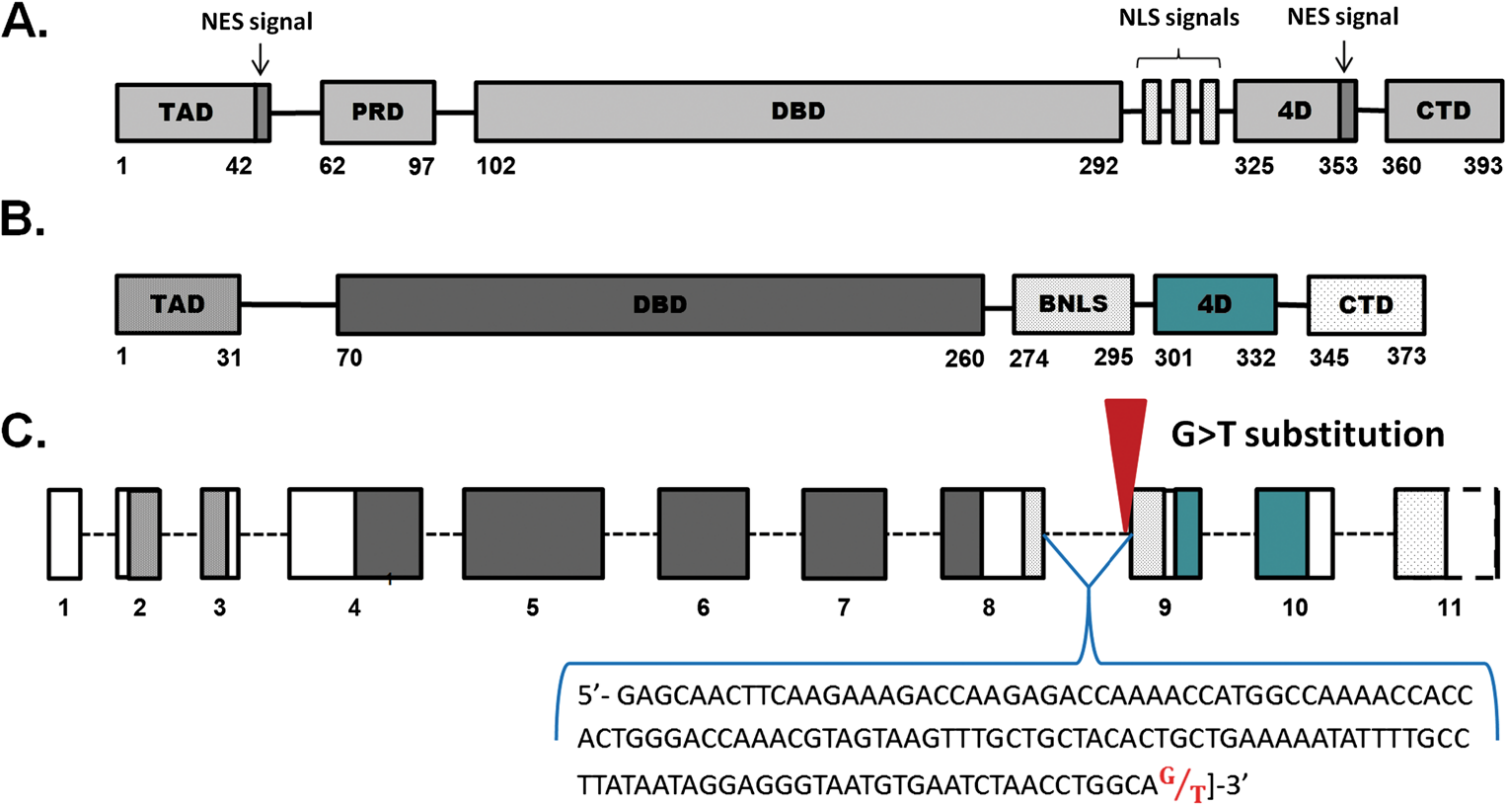
Scheme of the human (**a**) and the zebrafish (**b**) *p53* gene structure: an N-terminal transactivation domain (TAD), a proline-rich domain (PRD), a large DNA binding domain (DBD), a tetramerization domain (4D), a C-terminal regulatory domain (CTD), a nuclear export signal (NES), a nuclear localization signal (NLS), a bipartite nuclear localization signal (BNLS). Amino acid numbers for functional domains are indicated. (**c**) The *p53* ^hu888^ zebrafish line has a G to T point mutation at the splice-acceptor site in the intron 8 of *p53* gene. Numbers of constitutive exons are indicated

In humans, p53 is a 393 amino-acid protein composed of several domains: an N-terminal transactivation domain (TAD), a proline-rich domain (PRD), a large DNA binding domain (DBD), a tetramerization domain (4D), and a C-terminal regulatory domain (CTD). Nuclear export sequence (NES) signals exist on both the N- and C-terminals, whereas nuclear localization (NLS) signals are located on the C-terminal region (Fig. 1a). Interestingly, a high degree of conservation in the structure and function of p53 protein exists between distantly related organisms, such as fish and mammals (Brzuzan et al. 2009; Chen et al. 2009), reptiles and mammals (Soussi et al. 1987), and birds and mammals (Soussi et al. 1988). For example, the full-length amino acid sequence of zebrafish p53 is 48 % identical to human p53 (Cheng et al. 1997) and retains all important domains reported for humans, such as TAD, DBD, NLS, 4D, and CTD (Fig. 1b). Similarly, the *Xenopus laevis* p53 protein reveals a high degree of homology with the human (51 %) p53 amino acid sequence. Furthermore, five highly conserved internal regions were found in this protein (Soussi et al. 1987). The amino acid sequence of chicken p53 shows 47 % homology with human p53 (Soussi et al. 1988).

The principal mode of p53 alteration in human cancers are single-base substitutions in the *p53* coding sequence, leading to missense mutations, nonsense mutations or frameshifts (Olivier et al. 2010). More than 70 % of *p53* mutations are missense mutations affecting residues within DNA binding domain. In contrast, mutations in non-coding regions of *p53* have not been the subject of extensive study as compared to those performed on exons. In particular, mutations in intronic sequences which affect splicing sites, may potentially result in truncated protein products or reduced protein levels. To date, 29 different *p53* splice site mutations have been described in various types of cancers (Holmila et al. 2003). For example, an A>G transition in intron 10 that eliminates a splicing acceptor site and causes a frameshift mutation, was recently reported in a pediatric adrenocortical tumor (Pinto et al. 2011).

In recent years, it has become possible to generate numerous mutations in various zebrafish genes, to illuminate functional diversity of multiple phenotypes. Since launching of the Zebrafish Genome Sequencing Project in 2001, the Sanger Institute (http://www.sanger.ac.uk/) has played the lead role in sequencing the zebrafish genome, mapping variants, generating mutants, and characterizing them. A project called The Zebrafish Mutation Project (ZMP), which aims to create a knockout allele in every protein-coding gene in the zebrafish genome, using a combination of whole exome enrichment and Illumina next generation sequencing. So far, 11,892 genes have been mutated. Upon request, the ZMP provides zebrafish embryos carrying point mutations in *p53* gene. The available lines include embryos sa20404, sa20405, sa114, or hu888. While the first two mutant zebrafish carry nonsense mutations, the remaining two carry splice site mutation (http://www.sanger.ac.uk/Projects/D_rerio/zmp/).

The experimental power of the zebrafish p53 mutant line hu888 has been formerly indicated in the Zebrafish Mutation Project, however it has not been previously evaluated. The present study demonstrates the functional importance of this mutation in the *p53* gene in zebrafish. The *p53* ^*hu888*^ zebrafish mutants carry a mutation at position 25762707 of the chromosome 5 (GenBank accession no: NC_007116). This mutation is a substitution at the splice-acceptor site in the intron 8 of *p53* gene (Fig. 1c); it can cause intron retention, skipping of the succeeding exon or the use of a cryptic splice site, that is present within the exon downstream of the splice-acceptor site. Here, by evaluating sequencing data we demonstrated that both the constitutive and the next available legitimate splice site in exon 9 (the cryptic splice site) were used, and that the alternative splicing event resulted in a deletion of 12 nucleotides in the mature mRNA. Together with the DNA and mRNA data, we report protein levels of p53 in liver to assess their potential contribution to the molecular mechanisms of the mutant zebrafish.

## Materials and methods

### Zebrafish maintenance

The zebrafish p53 mutants (*p53* ^*hu888*^) were shipped from the Derek Stemple Lab (Welcome Trust Sanger Institute, Cambridge; UK). The delivered outcross stock (approx. 4 dpf larvae; parental generation, the P-stock) was obtained from spawning heterozygous mutant fish (*p53* ^*hu888/+*^) with the wild-type main line (*p53* ^*+/+*^), thus theoretically the progeny consisted of 50 % of mutation carriers and needed further identification. The obtained larvae were kept in 1 L baby tanks at 28.5 °C for their first 21 days and then raised in 20 L aquaria with aerated tap water at 28 °C and 14:10 photoperiod. The fish were fed 2-times a day with artificial Artemia and fish feed micropellets (AZOO; UK); the fish were maintained according to the procedures described in The Zebrafish Book (Westerfield 2000). After reaching sexual maturity (12 weeks), randomly selected individuals from the P outcross stock were separated into individual 5 L aquaria prior to the molecular identification.

### PCR-based identification of mutation carriers (p53^hu888^)

Caudal fin clips excised from anesthetized zebrafish were used for genomic DNA (gDNA) extraction with Genomic Mini kit (A&A Biotechnology; Poland). Zebrafish *p53* fragment containing wild type (wt) or mutant allele (hu888), was amplified using primers (Dr-p53-7266F: 5′-cag gaa aac tga gga gag caa ctt-3′ and Dr-p53-7502R: 5′-aga tct cct cgc tgc tgg-3′) that were positioned in 8 and 9 exon of zebrafish *p53*, respectively (GenBank accession no: NC_007116). Each PCR reaction contained 5 μL of 2X DreamTaq Green PCR Master Mix (Fermentas; Canada), 0.5 μM of each (forward and reverse) primer, 1 μL of gDNA as template, and nuclease-free water to 10 μL of the final volume. The reaction was performed in Master Cycler Gradient thermalcycler (Eppendorf; Germany) with the following conditions: 95 °C for 2 min, followed by 35 cycles of 95 °C for 30 s, 60 °C for 30 s, 72 °C for 30 s, and then 72 °C for 7 min. Amplified PCR product were separated in 1.5 % agarose gel and stained with ethidium bromide (EtBr) using standard protocol (Sambrook and Russell 2001). Homogenous amplicons, visualized as single bands of approximately 240 bp, were subjected to sequencing procedure (Genomed; Poland).

### Breeding of mutants for F1 generation

The heterozygous hu888 zebrafish (*p53* ^*hu888/+*^) were used to obtain offspring (F1) that theoretically should have consisted of 25 % of wild-type fish, 50 % of heterozygotes, and 25 % of hu888 homozygotes. For breeding purposes, modified method for low-level embryo production was used (Westerfield 2000). Briefly, 2 L plastic tanks with net instead of the bottom (mesh φ 3 mm) were immersed in 20 L glass tanks with tap water (28 °C and 14:10 photoperiod). During the experiment, spawning of *p53* ^*hu888/+*^ zebrafish was performed 14 times. Heterozygous fish were mated in breeding tanks at a ratio of 1 male to 2 females. The day after spawning, 30 min after the start of photoperiod, the zebrafish eggs were collected and rinsed with egg water (60 mg of Instant Ocean salts per L of distilled water (Westerfield 2000)). The mutant zebrafish exhibited sporadic spawning activity but successful in vitro fertilization. The average number of eggs laid per mating event was 199 (Witkowski 2013). The obtained zebrafish embryos (F1) were kept in 120 mm petri dish plates (25 embryos per dish) and sorted out for any coagulated eggs until hatching, with daily exchange of egg water. For all but one spawning the clutches were small (few individuals only) and died soon after hatching. Pericardial edema and jaw malformations occurred with higher incidence in some of the hu888 clutches. The hatched larvae were maintained as mentioned above. In one of the *p53* ^*hu888/+*^ clutch only, 19 zebrafish (11 males, and 8 females) out of 112 hatched, reached sexual maturity, and these fish (10-month old) were further subjected to molecular identification of their genotype using the same PCR-based method, as described above. Of the 19 zebrafish assayed, 12 were hu888 homozygotes (*p53* ^*+/+*^) and the remaining seven (five males and two females) were heterozygous (*p53* ^*hu888/+*^).

### RNA extraction, reverse transcriptase PCR, cloning, and sequence analysis

Total RNA from liver of wild type zebrafish (*p53* ^*+/+*^; *n* = 3) and hu888 heterozygotes (*p53* ^*hu888/+*^; *n* = 3) was isolated using Total RNA Mini isolation kit (A&A Biotechnology; Poland) following the manufacturer’s protocol; homozygous mutants were not identified in F1 progeny. As an additional control, the total RNA was isolated also from liver of wild type Tübingen zebrafish strain (“T” *p53* ^*+/+*^; *n* = 3) (Max-Planck Institute; Germany). The RNA was further treated with DNase I (Fermentas; Canada) to remove gDNA. The quality and quantity of the mRNA samples were measured using the Bioanalyzer 2100 (Agilent; USA). The DNase- treated RNA was converted to cDNA using the Revert Aid First Strand cDNA Synthesis Kit and oligo-dT primer (Fermentas; Canada). The cDNA was then used as a template for amplification of *p53* fragments using 12.5 μL of DreamTaq Green PCR Master Mix (Fermentas; Canada), 0.5 μM of each primer (Dr-p53-7266F, Dr-p53-7502R), 2 μL of cDNA as template, and nuclease-free water to 25 μL of the final volume. The PCR reaction was performed in Master Cycler Gradient thermalcycler (Eppendorf; Germany) by using the following thermal cycling program: initial denaturation at 95 °C for 3 min; 35 cycles consisting of denaturation at 94 °C for 30 s, annealing at 60 °C for 30 s, and amplification at 72 °C for 30 s; and a final 7-min hold step at 72 °C. The corresponding controls lacking reverse transcriptase or template RNA were included for each reaction to confirm the absence of genomic DNA. PCR products were separated by electrophoresis (6.2 V/cm for 2 h) in 3 % low melting agarose gel containing EtBr. Furthermore, separated amplicons (visualized as single bands at respective lengths of 152 and 164 bp) were excised from the gel, then melt at 50 °C and used in reamplification using the same PCR components and thermal conditions. Reamplified PCR products were cloned into pTZ57R/T vector using InsTAclone PCR Cloning Kit (Thermo Scientific; Canada) according to the manufacturer’s recommendations. At least two independent clones of each amplicons were sequenced in both directions (Genomed; Poland). Amplified PCR products were sequenced to reveal splicing events of zebrafish *p53*.

### Size determination of the different PCR products corresponding to p53 variants

Each cDNA sample was also amplified using a forward primer labeled at 5′ end with 6FAM dye (Dr-p53-7267F_FAM (5′- gaa aac tga gga gag caa ctt-3′) from Applied Biosystems; UK, and Dr-p53-7502R (5′-aga tct cct cat cgc tgc tgg-3′) (for PCR conditions see above). Lengths of PCR fragments were determined using an Applied Biosystems 3130 Genetic Analyser sequencer against GeneScan 500[LIZ] (Life Technologies, USA) size standard. The separation of PCR product was performed in capillary array at length of 36 cm and POP7 polymer following to manufacture’s recommendations. The product’s size determination was performed using Data Collection 3.0 software and GeneMapper 4.0 software (Life Technologies; USA), following the manufacturer’s recommendations.

### SDS-PAGE and western blot analysis

Livers from wild type zebrafish (*p53* ^*+/+*^), wild type Tübingen zebrafish strain (“T” *p53* ^*+/+*^) and hu888 heterozygotes (*p53* ^*hu888/+*^) (*n* = 3 in each group) were lysed in RIPA buffer containing protease inhibitor cocktail set (Calbiochem; USA). Lysates were centrifuged at 10,000 g for 20 min at 4 °C to remove cell debris and insoluble proteins. After centrifugation, the supernatant was transferred into a chilled Eppendorf tube. Protein extract were quantified by Bradford assay and equal amounts (20 μg) of protein was separated by a 10 % SDS-PAGE. After electrophoresis, the proteins were electrotransferred onto a PVDF membrane (Sigma Aldrich; USA) using a Mini Trans-Blot electrophoretic transfer cell (Bio-Rad; USA) at 200 mA for 1 h. Nonspecific protein binding was blocked with 5 % BSA in 1X TBST (50 mM Tris–HCl pH 7.4, 150 mM NaCl, 0.1 % Tween 20) for 1.5 h at room temperature. The membrane was then incubated overnight at 4 °C with rabbit anti-zebrafish p53 antibody (#55342, Anaspec, San Jose; CA), and subsequently incubated with HRP-conjugated goat anti-rabbit IgG antibody (Cell Signaling; USA) for 1 h at room temperature. The blots were subsequently probed with anti-actin antibody (sc-1616-R, Santa Cruz Biotechnology; USA) to assess protein loading. All antibodies were diluted in 1X TBST at a concentration 1:1000. Antibody labeling was identified using 4-chloro-1-naphthol/3,3́- diaminobenzidine tetrahydrochloride (CN/DAB, Thermo Scientific; Canada) as substrates.

## Results and discussion

The hu888 zebrafish line was established under the Zebrafish Mutation Project with G to T point mutation at the splice-acceptor site in the intron 8 of *p53* gene, likely rendering the mutant *p53* gene a target of alternative splicing (Fig. 1c). Only wild type zebrafish (*p53* ^*+/+*^) and fish bearing a single mutant hu888 allele (heterozygotes; *p53* ^*hu888/+*^) survived to adulthood, with no consistently observed abnormalities during development. The lack of survival of homozygous hu888 carriers is intriguing and suggest functional insufficiency of resulting p53 phenotype during development. Further studies should focus on cellular effects caused by aberrant proteins encoded by the pair of two defective alleles. Fin malformations were observed sporadically in some adult fish, but did not appear to cause significant morbidity. The heterozygous hu888 zebrafish was characterized by occasional spawning activity, successful in vitro fertilization but high larval mortality; most of the hatched zebrafish did not survive 48 h after the hatching. Although, the above characteristics may suggest that the single hu888 mutation does not prevent reproductive function in this line, irregular spawning may indicate deficits in reproductive physiology or behavior.

Splice site mutations constitute the most common cause for alternative splicing. Splicing occurs in several steps and is catalyzed by small nuclear ribonucleoproteins (snRNPs). The three sequence elements that direct this RNA processing include a donor site (5′ end of the intron) with an almost invariant sequence GU, a branch site (near the 3′ end of the intron) and an acceptor site (3′ end of the intron) includes an almost invariant dinucleotide AG. First, the 5′ splice site is cleaved and the 5′ end of the intron is joined to conserved branch site to form a looped structure known as a lariat. The first 9 nt of the 5′ end of the snRNP called U1 is complementary to the consensus sequence 5′-C(or A)AG GU(A)(or G) AGU-3′ (natural 5′ splice site sequences usually deviate from this consensus sequence at two or three positions) (Clancy 2008). Base pairing of the U1 snRNP and the mRNA is essential for splicing. The G>T mutation at the splice-acceptor site causes a mismatch with the 3′ end of the U1 snRNP. This type of mutation can lead to intron retention, skipping of the succeeding exon, or activation a nearby cryptic splice-acceptor site, that is present within the coding region. The cryptic splice-acceptor site is located presumably by a scanning mechanism that initiates at the branch site and proceeds in a 3′ direction to the first AG pair (Smith et al. 1993).

**Fig. 2:**
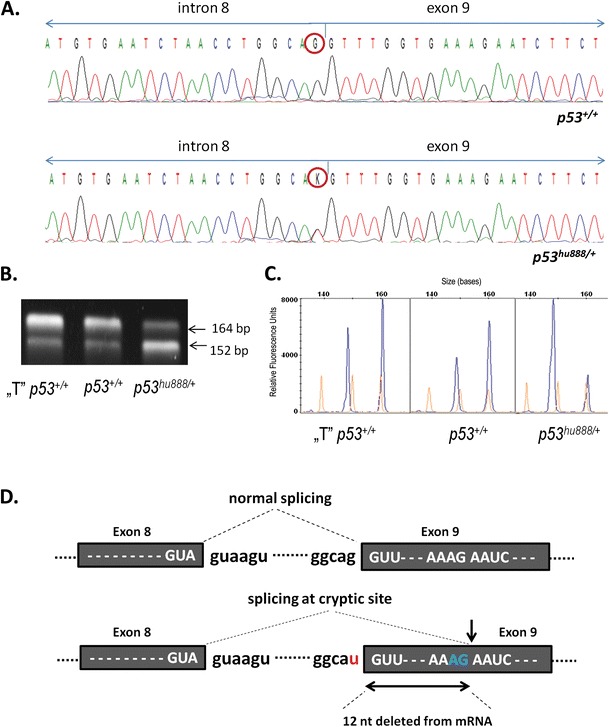
Expression of p53 splicing variants in hu888 zebrafish line. (**a**) Electropherograms of DNA sequences spanning regions of the G/T mutation (K). (**b**) Agarose gel electrophoresis of RT-PCR amplification of mRNA from liver of two wild type zebrafish line and *p53* ^hu888^ zebrafish mutant (heterozygous). Primers Drp53- 7266F and Dr-p53-7502R were used. Lane “T” *p53*: amplicon of the mRNA expressed from the liver of wild type Tübingen zebrafish strain, lane *p53*: amplicons of the mRNA expressed from the liver of wild type Sanger zebrafish strain, lane *p53* ^hu888/+^: amplicons of the mRNA expressed from the liver of p53hu888 zebrafish mutant (heterozygous). Normally spliced mRNA yielded a 164-bp amplicon, whereas the alternative splicing resulted in a 152-bp amplicon. (**c**) Expression intensities of *p53* splice isoforms in liver of two wild type zebrafish line and *p53* ^hu888/+^ zebrafish (blue peaks); size standard GS 500LIZ (orange peaks). (**d**) Splicing mechanism using a normal splice site (above). The resulting product corresponds to the bands of 164 bp. Alternative splicing mechanism using a cryptic splice sites (below), owing to mutations at splice-acceptor site G>T (marked in red). In case of this abnormal splicing pathway 12 nt are deleted from downstream of exon 9. The resulting product corresponds to the bands of 152 bp

To find out whether G>T substitution at the splice-acceptor site in the intron, between exon 8 and exon 9 of the *p53* (Figs. 1c and 2a), influenced the processing of the RNA transcript, we performed reverse transcriptase-polymerase chain reaction (RT-PCR) on total cDNA, prepared from liver from two wild type zebrafish line (“T” *p53* ^*+/+*^ and *p53* ^*+/+*^) and from mutant zebrafish (*p53* ^*hu888/+*^). Gel electrophoresis of resulting PCR products showed two amplicons differing in length (Fig. 2b and c). The longer amplicon of about 164 bp corresponds with a predicted PCR product resulting from constitutive mRNA splicing, whereas the shorter amplicon of about 152 bp was apparently a result of alternative splicing. Sequencing of the shorter amplicon revealed that a cryptic splice-acceptor site within exon 9 was used, resulting in the deletion of 12 nt from the 5′ end of exon 9 in the p53 mRNA (Fig. 2d), and the sequence of the cryptic splice-acceptor site was AAG|AAUC.

Interestingly, the constitutive (164 bp) and alternative (152 bp) splice variants of *p53* gene were present in two wild type zebrafish line and in mutant hu888 zebrafish (Fig. 2b and c), but differed in the amount of the respective splice variant: while constitutive splicing within intron 8 occurred more frequently in p53 wild type lines (Tübingen, hu888) than activation a cryptic splice site in exon 9, the alternative splicing was a predominant process in heterozygous zebrafish (*p53* ^*hu888/+*^). This proves that cryptic splice sites are used not only when a constitutive splice site is disrupted by a mutation. In human, spliced isoforms are frequently regulated in a developmental or tissue-dependent manner (Wang et al. 2008; Xu et al. 2002). Moreover changes in the ratios of isoforms have been associated with physiological variation in mammals and their susceptibility to disease (Chisa and Burke 2007; Cooper et al. 2009). Therefore, further research should focus on tracing co-occurrence of the two splice variants in zebrafish during development which may shed light on mechanisms regulating expression of p53 isoforms in vertebrates.

Besides full-length p53 protein, various p53 isoforms are produced by alternative splicing, alternative promoter or codon initiation usage, or combinations thereof (Marcel et al. 2011). The resulting proteins differ from canonical, full-length p53 protein, by truncation of a variable portion of the N-terminus (ΔN isoforms) and by alternative C-terminal portions (C-terminal isoforms) (Bourdon et al. 2005; Marcel et al. 2011). So far, at least 12 different p53 human protein isoforms have been identified (Khoury and Bourdon 2010). The differential expression of several of these isoforms has recently been established in cancer (Bourdon 2007; Wei et al. 2012) though their functional role is not fully understood. To date, in zebrafish only N-terminal p53 isoforms have been identified: Zp53, corresponding to the human p53 protein (Cheng et al. 1997), ZΔNp53 produced through an alternative splicing of intron-2 (Davidson et al. 2010) and ZΔ113p53, produced by an internal promoter located within the zebrafish *p53* gene that is regulated by p53 itself (Chen et al. 2005).

**Fig. 3:**
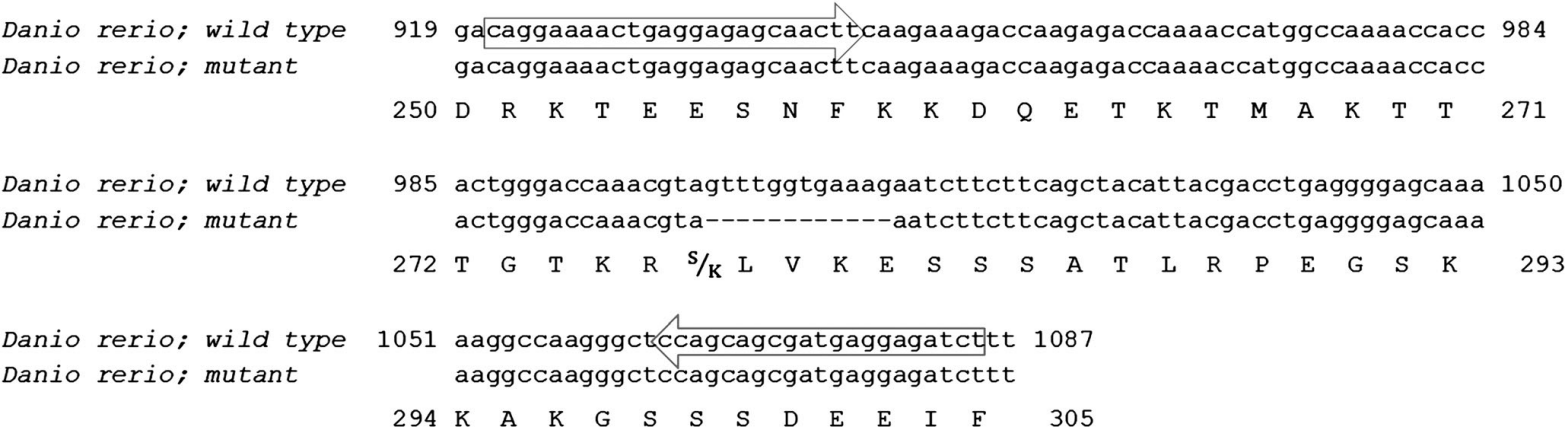
Clustal X alignments of two p53 splicing products obtained in this study. The nucleotide sequences of p53 amplicons from both wild type (*p53* ^+/+^) and *p53* ^hu888^ zebrafish. The RT-PCR primers are indicated by arrows. The amino acid sequence of zebrafish is shown (NCBI: NP_001258749.1)

**Fig. 4:**
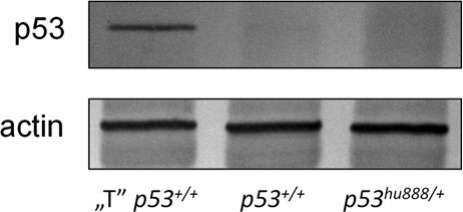
Representative Western blot results from Western blot analysis of p53 expression in two wild type zebrafish lines and *p53* ^hu888/+^ mutant. Actin was used as the loading control. “T” *p53*: wild type Tübingen zebrafish strain; *p53*: wild type Sanger zebrafish strain; *p53* ^hu888/+^: *p53* ^hu888^ zebrafish mutant (heterozygous)

Deletion of 12 nucleotides from a mature p53 mRNA, as a consequence of G>T substitution at splice site, results in four amino acid removal in the encoded zebrafish protein, from leucine-278 to glutamic acid-281 and with replacement serine by lysine at 277 position (Fig. 3) without changing the reading frame. Further bioinformatics analysis of the isoform localized the deletion to a domain specifying a putative bipartite nuclear localization signal (BNLS) (Fig. 1b and c). Bipartite localization signal controls the p53 protein shuttling between the cytoplasm and the nucleus which is crucial for its tumor suppressive activity (Sionov et al. 2001) and it has been found that point mutations in the *p53* NLS can severely reduce activity of p53 nuclear import (O’Keefe et al. 2003). To find out if zebrafish having partially deleted domain in putative BNLS of the p53 isoform (*p53* ^*hu888/+*^) exhibits abnormal p53 expression we examined the p53 levels in livers of the three zebrafish lines. The observation of both, *p53* ^*hu888*^ mutant and p53 wild type hu888 zebrafish having highly reduced levels of p53 in liver compared to wild type Tübingen strain (Fig. 4), excluded the possibility that hu888 mutation itself is responsible for the apparent reduction effect. One explanation for the faint bands of p53 observed in hu888 zebrafish could be the inability of the antibody to recognize the mutant isoform as a result of truncation in the p53. To demonstrate a consequence of the loss of nuclear localization, assay of the expression of transcriptional targets of p53, such as p21, should be performed (e.g., Rose et al. 2003). Additionally, cellular experiments in which tagged constructs of the truncated version are injected into zebrafish would probably clarify this issue (Rosen et al. 2009). It is also likely that the mutant line hu888 carries other mutations throughout the *p53* gene, which result in aberrant expression (attenuation) of p53 protein, however further studies are necessary to clarify this issue.

The above data suggest that intronic G>T mutation has little effect on the reduction of a cell’s overall p53 concentration in heterozygous zebrafish, and it is unlikely that it significantly disturbs p53 function under normal conditions. It remains unknown however, what effect the aberrant p53 protein may have on successful executing defense programs in response to cell physiological signals typically provoking p53 increases. The G>T transversion, and other mutations, in *p53* gene of hu888 zebrafish line may be considered dominant negative (dn) mutations whose altered gene products act antagonistically to the wild-type allele (Goh et al. 2011). The p53 protein normally functions as a homotetrameric transcription factor. However, in cells bearing a single mutant *p53* allele that codes for a structurally altered protein, the mutant protein may retain its ability to form tetramers but may lose its ability to exert normal function. Consequently, mixed tetramers composed of differing proportions of wild-type and mutant p53 subunits may form, and thus may compromise the functioning of the entire tetramer; in a cell that is heterozygous at the *p53* locus, like that in hu888 zebrafish line, as many as 15 of 16 of the subunits may lack fully normal functions. Therefore, future experiments on the hu888 zebrafish line are needed to resolve how the *p53* mutant isoforms interfere with wild type *p53*. This would allow to gain further insight into role of the p53 isoforms in embryo development, cancer and aging.
